# A Case Series of Psychological Stress Evaluation as a Risk Factor for Oral Lichen Planus

**DOI:** 10.1155/2022/1915122

**Published:** 2022-10-06

**Authors:** Irna Sufiawati, Ani Megawati, Muhammad Al Farisyi, I. Nyoman Gede Juwita Putra

**Affiliations:** ^1^Department of Oral Medicine, Faculty of Dentistry, Padjajaran University, Indonesia; ^2^Oral Medicine Residency Program, Faculty of Dentistry, Padjadjaran University, Indonesia; ^3^Oral Medicine Clinic, Kota Prabumulih General Hospital, South Sumatra, Indonesia; ^4^Department of Oral Medicine, Faculty of Dentistry, Mahasaraswati University, Indonesia

## Abstract

**Introduction:**

Oral lichen planus (OLP) is a chronic inflammatory disease of the oral mucosa with unknown etiology. Suggested predisposing factors for the development of OLP include genetic factors, viral infections, psychological stress, trauma, drug intake, and some systemic diseases. This serial case aimed to evaluate the psychological stress in triggering various types of OLP and its management. *Case Report*. Six patients, four females and two males with an age range from 23 to 57 years, came to an oral medicine clinic with chief complaints of chronic pain and burning sensations in the oral cavity. All cases showed typical clinical features of OLP on the oral mucosa, including reticular, plaque-like, erosive, and ulcerative lesions. An incisional biopsy was performed in some cases and the histopathology features confirmed the diagnosis of OLP with no signs of dysplasia/malignancy. According to the Depression, Anxiety, Stress Scale (DASS)-21, the patients had different levels of depression ranging from mild to severe; 3 patients were considered to have moderate anxiety and others had severe anxiety; and 5 patients experienced mild stress and 1 patient had moderate stress. The patients were given corticosteroids, supportive therapy, and psychological counseling. All patients experienced significant improvement of the lesions after treatment.

**Conclusion:**

Psychological stressors play an essential role as a risk factor in the development of OLP. Therefore, management of OLP encompasses not only an appropriate treatment of the lesions but also psychological intervention.

## 1. Introduction

Oral lichen planus (OLP) is a chronic inflammatory disease of unknown etiology affecting the oral mucosa. The estimated prevalence of OLP in adults is roughly 0.5–2%. The ratio for this disease in males and females is 2 : 1 and the average age of onset is 30–60 years old [1, 2]. OLP is a potentially malignant disorder despite the lingering controversy regarding the potential of OLP malignant transformation; however, the chance of OLP malignant transformation is only 0–12.5% [3–5]. The diagnosis of OLP is usually made by clinical and histological examinations. The clinical features of OLP are bilateral with symmetrical patterns and distinctive buccal mucosa. Other commonly affected sites are the tongue, gingiva, and labial mucosa [6]. On the other hand, an uncommon OLP lesion may also affect the palate, floor of the mouth, and lips. There are six clinical subtypes of OLP that can be observed: reticular, plaque-like, atrophy, erosive/ulcerative, papule, and bullous. The most common subtypes of OLP are reticular, erosive/ulcerative, and plaque-like [7, 8]. The risk factors of OLP are genetic, viral infections, psychological stress, trauma, and the association of systemic diseases that often be one of the complaints in OLP patients [9, 10].

Psychological stressors have been shown to be contributory to the pathogenesis of OLP since OLP patients often experience depression and anxiety [1, 2, 11–13]. Many studies suggest that psychological stress can alter the innate and adaptive immune systems. These alterations are primarily mediated by a neuroendocrine mediator from the hypothalamic-pituitary-adrenal (HPA) and sympathetic adrenal axis, through peripheral neuropeptide release and the sympathetic nervous system [14]. Thus, stress and anxiety can be some of the risk factors for OLP.

The purpose of this case report is to evaluate psychological stress as a possible trigger of OLP and a multidisciplinary therapeutic approach in these presented cases.

## 2. Case Report

### 2.1. Case 1

A 42-year-old man visited the oral medicine clinic with a complaint of painful ulcers on the right and left inner cheeks for the last 3 months. He has no history of any systemic disease, but he was often stressed by his job as a contractor. The patient had visited a dentist and was prescribed aloe vera mouthwash and lip ointment, but there was no improvement. The patient stated that he was quite stressed because of his job.

**Figure 1:**
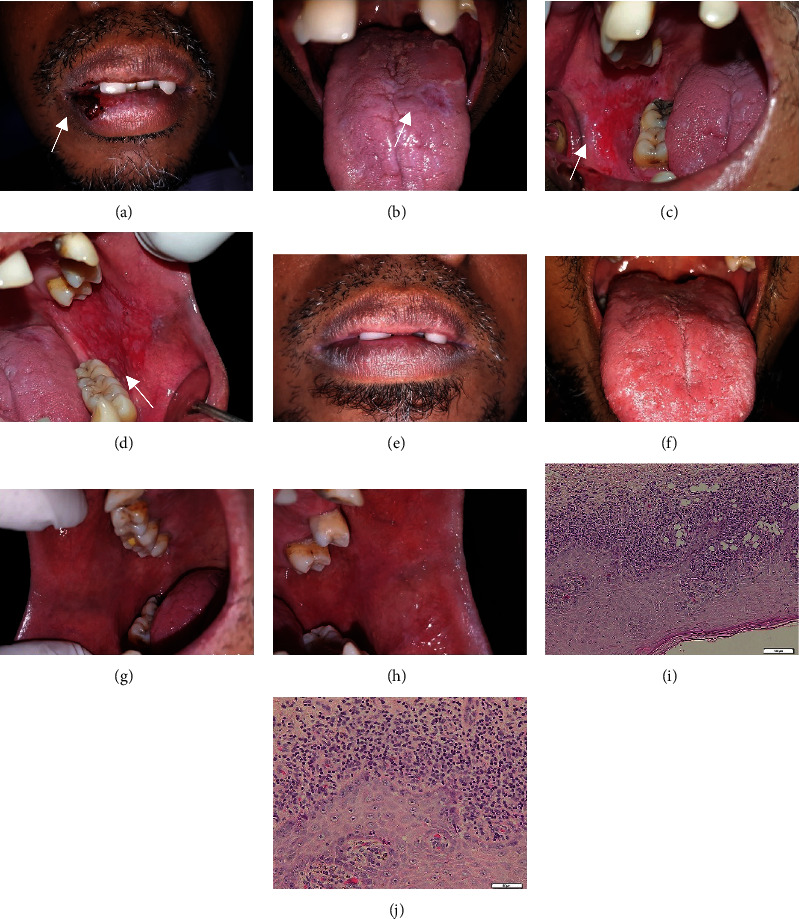
The clinical features of OLP in a 42-year-old man; (a) hemorrhagic crust, erosive surrounded by radiating white striae (Wickham striae) on the lower lip; (b). Erosive lesion surrounded by Wickham striae on the left dorsum of the tongue; (c) and (d). Erosive and ulcerative lesions on the right and left buccal mucosa; (e)–(h) Lesions on the lips, dorsum of the tongue, and the buccal mucosa completely healed after two months of treatment; (i) and (j) Histopathological features of the lesion with 100x and 400x magnification.

Extraoral examination showed hemorrhagic crust, erosive lesions surrounded by radiating white striae (Wickham striae) on the lower lips. Intraoral examination showed an erosive lesion with radiating white striae (Wickham striae) on the dorsum of the tongue, and also erosive/ulcerative lesions on the right and left buccal mucosa. Based on the clinical features, the suspected diagnosis for this case was the reticular, ulcerative, and erosive type of OLP (Figure 1(a)–1(d)).

A depression test was carried out using the Depression, Anxiety, and Stress Scale (DASS)-21 questionnaire. The result of the test showed that the patient was in a state of moderate depression, severe anxiety, and mild stress (Table 1). An incisional biopsy from the right buccal mucosa was performed to confirm the diagnosis. The histopathological features of the lesion showed a layer of stratified squamous epithelium that underwent a lichenoid reaction in the form of damage to basal cells and interface dermatitis. In the subepithelial cells, a massive inflammatory cell of lymphocytes, plasma cells, eosinophils, and histiocytes along the upper lamina propria can be seen forming a band-like pattern. The melanin pigment can also be seen. There was no sign of malignancy. These histopathological findings are in line with OLP (Figures 1(i)–1(j)).

The patient was administered moderate systemic corticosteroid (prednisone 30 mg/day for 14 days and then tapered), supportive therapy (multivitamin), and oral hygiene instruction (OHI). The patient was also referred to a psychological clinic to manage the stress level. In two months, the prednisone dose was reduced after each visit. Moreover, the pain in the oral cavity disappeared and the patient was able to eat normally. Intraoral examination showed that all lesions were improved after two months (Figures 1(g) and 1(h)). No pain or other symptoms were present. The patient has no longer felt as anxious as before. He was recalled and psychological tests were performed; the DASS-21 results showed decreasing scores of depression and anxiety (Table 1).

### 2.2. Case 2

**Figure 2:**
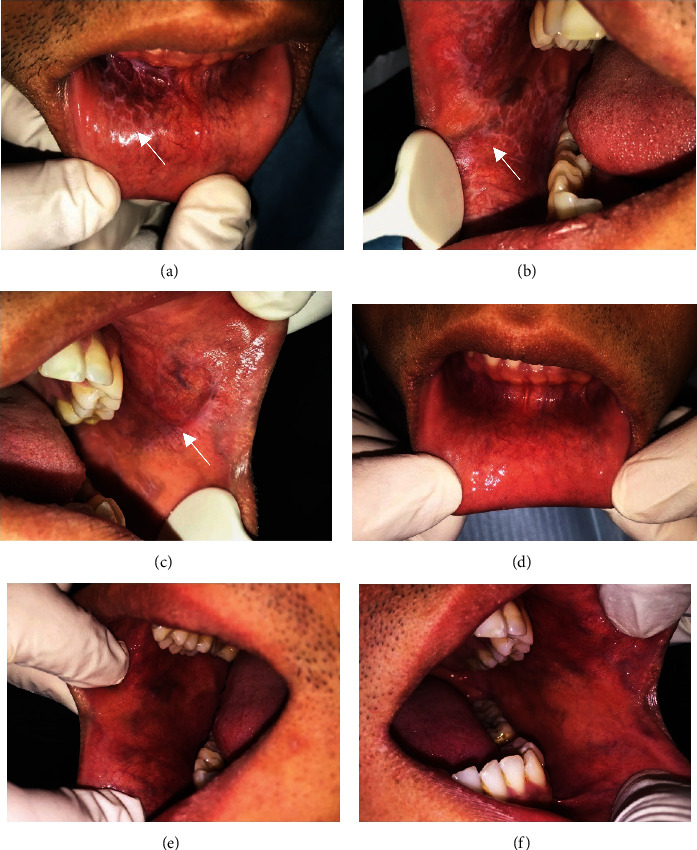
The clinical features of OLP in a 31-year-old man; (a)–(c). Wickham striae on the lower labial mucosa and on the right and left buccal mucosa; (d)–(f). Oral lesion completely healed after five months of treatment.

A 31-year-old man complained of ulcer and discomfort when eating for the last three months. The man had visited several doctors and was prescribed antibiotics, analgesics, and mouthwash, but there was no improvement. The patient admitted to having family and work issues for the past few months. On intraoral examination, we found characteristics of Wickham striae with erosive areas on the lower labial mucosa and on both sides of buccal mucosa (Figures 2(a)–2(c)). The results of the DASS test revealed a severe score of depression and anxiety and a moderate level of stress (Table 1). There was no histopathological examination because the patient refused to get a biopsy. Based on the clinical examination, the patient was diagnosed with OLP with erosive and reticular type. The patient was initially administered moderate systemic corticosteroid (30 mg prednisone/day orally) for 2 weeks, multivitamins, and OHI. The patient was also referred to a psychologist for stress management. At the second visit, the lesions had subsided, and the patient was given a combination of lowered-dose prednisone and azathioprine. The oral lesion was completely healed after five months of treatment (Figures 2(d)–2(f)). The patient felt comfortable while eating and no longer felt depression after five months. We recently recalled the patient to do a psychological test using DASS-21, and the results showed that the levels of depression and anxiety were reduced compared to the first visit (Table 1).

### 2.3. Case 3

**Figure 3:**
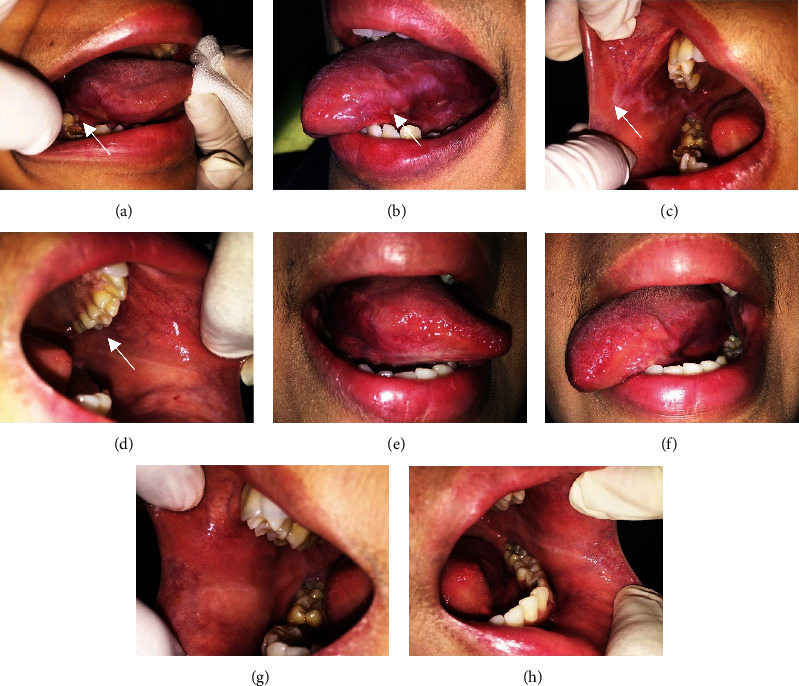
The clinical features of OLP in a 23-year-old woman; (a) and (b) yellowish pseudomembranous on the ventral of the tongue; (c) and (d) white plaques with striae surrounded by erythematous on the right and left buccal mucosa; (e)–(h) Oral lesion showed a significant improvement after two months of treatment.

A 23-year-old woman complained of a painful ulcer on her tongue that caused discomfort while eating for the last three weeks. The patient had consulted a dentist and was prescribed aloe vera mouthwash, but no improvement could be seen. The patient admitted to having work issues for the past few months. Intraoral examination revealed a yellowish pseudomembranous surrounded by a well-defined erythematous with irregular borders on the ventral side of the tongue (Figures 3(a) and 3(b)). White plaques with striae surrounded by erythematous can be seen on the left and right buccal mucosa (Figures 3(c) and 3(d)). The DASS test results revealed that the patient was considered to have moderate depression, severe anxiety, and mild stress (Table 1). Histopathological examination was not carried out since the patient had refused to do a biopsy. Based on the clinical examination, the patient was diagnosed OLP with reticular and erosive type. The patient was treated with systemic corticosteroid, supportive therapy, and OHI. The patient was also referred to a psychological clinic for stress management. The oral lesion showed a significant improvement within two months of therapy. After three months of treatment, the oral lesion completely healed (Figures 3(e)–3(h)). The patient has no complaint of pain while eating and no longer felt anxious as before. Recently, the DASS-21 was carried out again and the results showed that the levels of anxiety and stress were reduced (Table 1).

### 2.4. Case 4

**Figure 4:**
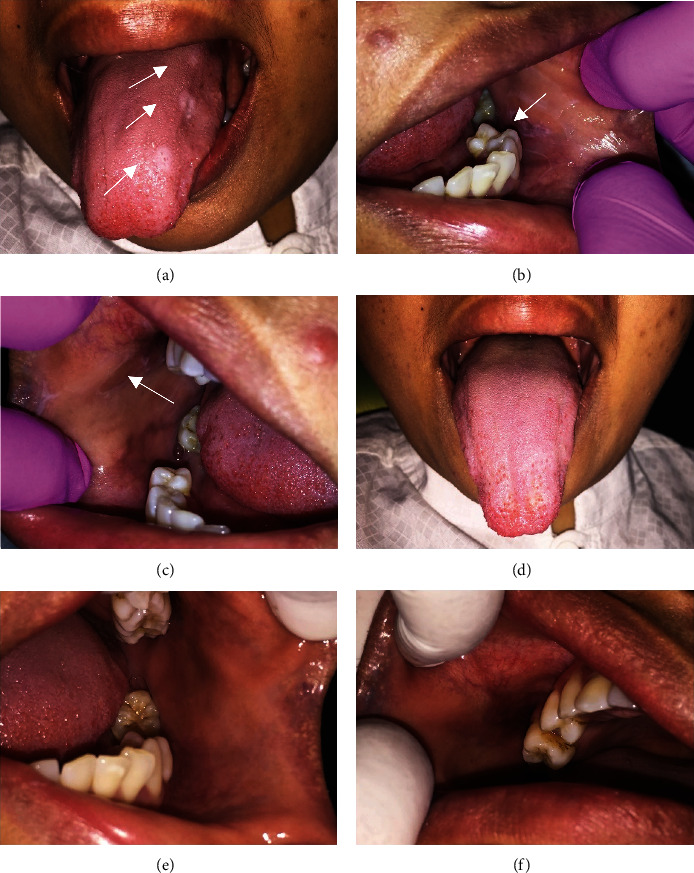
The clinical features of OLP in a 32-year-old woman; (a) multiple white plaques on the dorsum of the tongue; (b) and (c) erosive lesions surrounded by Wickham striae on the right and left buccal mucosa; (e)–(g) oral lesion was completely healed after three months of treatment.

A 32-year-old woman complained of white patches and numbness on her tongue. The patient also had a habit of smoking and using over-the-counter mouthwash regularly. The patient admitted to having business issues for the past few months. Intraoral examination revealed multiple round white plaques with clear and irregular borders on the dorsum of the tongue. White plaques with striae surrounded by erythematous could also be seen on the left and right buccal mucosa (Figures 4(a)–4(c)). The DASS test results revealed that the patient had severe depression, moderate anxiety, and moderate stress (Table 1). Histopathological examination was not carried out since the patient had refused to do a biopsy. Based on the clinical examination, the patient was diagnosed OLP with reticular and plaque-like type. The patient was administered corticosteroid mouthwash, supportive therapy, and OHI. The patient was referred to the Department of Psychology for stress management. After three months of therapy, the lesion showed a significant improvement (Figures 4(d)–4(f)). The patient felt no numbness of taste and was anxious anymore. The DASS-21 was done and the levels of depression were lower compared to the first visit (Table 1).

### 2.5. Case 5

**Figure 5:**
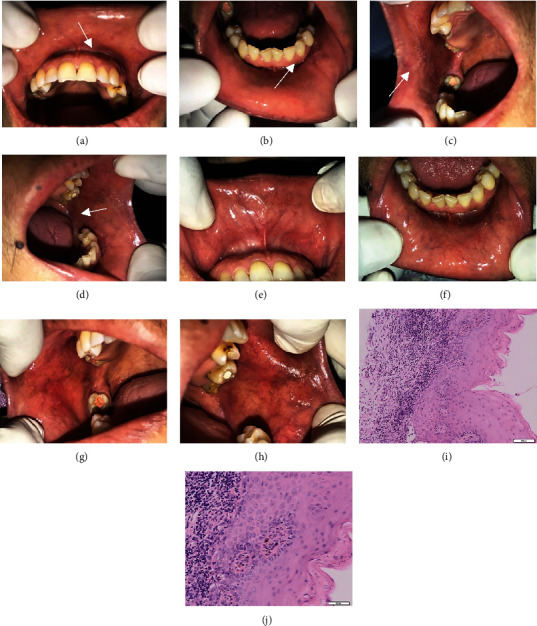
The clinical features of OLP in a 54-year-old woman; (a) and (b) erythema with radiating white striae on the upper and lower labial mucosa; (c) and (d) Wickham striae on the lower left labial mucosa and on the left buccal mucosa; (e)–(h) lesion on the upper and lower labial mucosa and the right and left buccal mucosa showed a significant improvement; (i) and (j) histopathological features of the lesion with 100x and 400x magnification.

A 54-year-old woman visited the oral medicine clinic with a complaint of pain and burning sensation throughout the oral cavity for the past three months. The patient also felt some discomfort when eating or drinking which resulted in lethargy and lack of motivation. The patient had no record of allergies. The patient admitted to feeling extremely anxious due to family issues for the past few months. Intraoral examination revealed white plaques on the right and left buccal regions which formed a bilateral pattern similar to Wickham striae with multiple erosive (Figures 5(a)–5(d)). The DASS-21 test revealed that the patient experienced mild depression, moderate anxiety, and moderate stress (Table 1). Histopathological examination showed the presence of stratified squamous epithelium with inflammatory cells in the epidermis and cell nucleus but still within normal limits. No signs of malignancy can be found (Figures 5(i) and 5(j)). Based on the clinical and histopathological examination, the patient was diagnosed OLP with reticular and erosive type.

The patient was administered systemic corticosteroid, supportive therapy, and OHI. The patient was also referred to a psychological clinic for stress management. The lesion on the labial mucosa dan buccal mucosa showed a significant improvement after two months of treatment (Figures 5(e)–5(h)). The patient has no longer burning sensation in her mouth and no longer felt anxiety as before. The DASS-21 was performed again. She was considered to have only mild anxiety, while the scores of depression and stress were reduced to normal levels (Table 1).

### 2.6. Case 6

**Figure 6:**
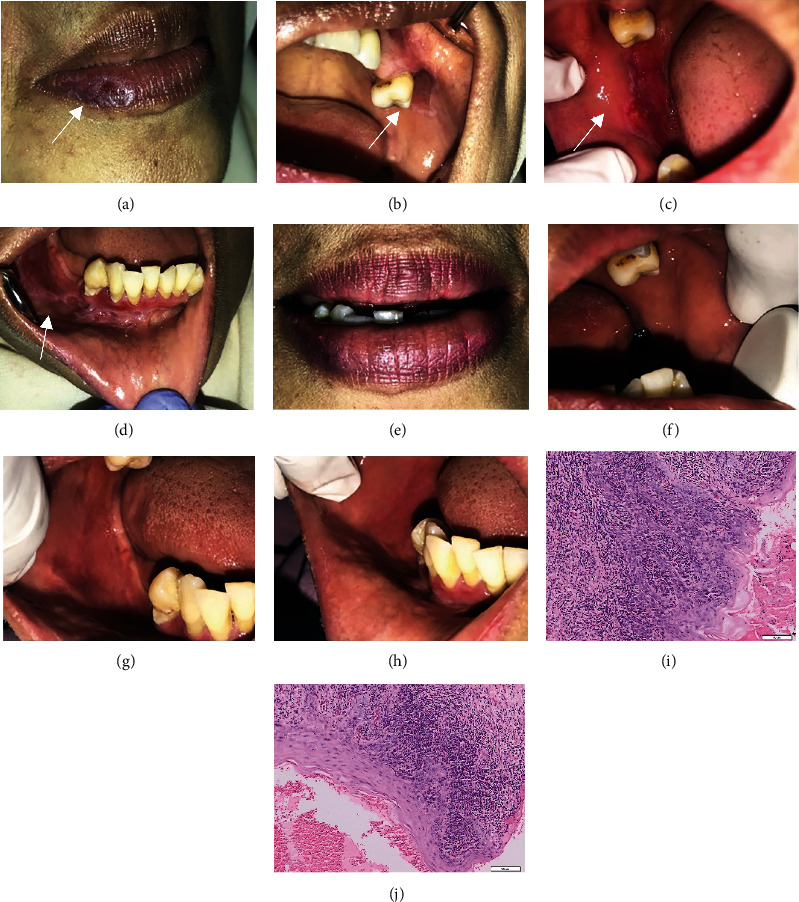
The clinical features of OLP in a 57-year-old woman; (a) erosive lesion and Wickham striae on the right lower lip; (b)–(d) bilateral erosive lesion surrounded by Wickham striae on the right and left buccal mucosa; (e)–(h) lesions showed improvement after 2 months of treatment; (i) and (j) microscopic appearance of the lesion with 100x and 400x magnification.

A 57-year-old woman visited the oral medicine clinic with complaints of pain and burning sensation throughout the oral cavity for the last four months. The patient complained that these conditions had worsened over the last few weeks. The patient was extremely uncomfortable with the conditions and believed that they were symptoms of oral cancer. The DASS-21 test results showed that the patient had mild depression, moderate anxiety, and mild stress (Table 1). Extraoral examination revealed the presence of Wickham striae on the right lower lip. Intraoral examination revealed bilateral white striae on the right and left buccal mucosa (Figures 6(a)–6(d)). Histopathological examination revealed hyperkeratosis of stratified squamous epithelium, but the nucleus was still within normal limits (Figures 6(i) and 6(j)). Based on the clinical and histopathological examination, the patient was diagnosed OLP with reticular and erosive type.

The patient was administered systemic corticosteroid, supportive therapy, and OHI. The patient was also referred to a psychologist for stress management. After two months, the pain and burning sensation in the oral cavity had disappeared. The patient was able to eat without any discomfort and no longer felt as anxious as before. Based on the intraoral examination, the Wickham striae on the right and left buccal mucosa had improved, leaving diffuse erythematous on the affected areas (Figures 6(e)–6(h)). Systemic corticosteroid was still administered at a lower dose. The DASS-21 was recently performed again and the results indicated that the levels of depression and anxiety were reduced to mild, but she still experienced mild stress (Table 1).

The characteristic of the patients, clinical profile of OLP, and their treatment were summarized in Table 2. The results of the DASS-21 test before and after the treatment can be seen in Table 1.

## 3. Discussion

Based on the six OLP cases above, all the patients experienced some sort of psychological stress. The DASS-21 scores revealed different levels of psychological stress for each patient. Stress was experienced due to family problems, work-related issues, pressure, fear of possible malignancy, and fear of unsuccessful treatments. Pain, anxiety, and fear can cause some changes in the metabolic and endocrine systems, causing physiological side effects such as increased cortisol. Cortisol is glucocorticoid 21-carbon secreted by the adrenal cortex to regulate the metabolism of protein, carbohydrate, fat, and water that can affect the nervous system sensitivity and stress responses [14, 15].

According to the relationship between stress and OLP, stressful life changes play an important role in the onset or reactivation of OLP, depending on each person's ability to cope and their particular reaction to stress [11]. Psychological conditions such as anxiety, depression, and stress tend to be more prevalent in OLP patients. Karthikeyan et al. (2016) found that serum, saliva, and urine cortisol levels in the OLP group were significantly higher than in the control group, indicating that the level of psychological stress was higher in the study group compared to the control group [12]. Based on these studies, it can be concluded that psychological stress is indicated to have a role in the manifestation of OLP.

The pathogenesis of OLP is quite complex. OLP is a cell-mediated autoimmune reaction in which CD4+ and CD8+ T lymphocytes induce the apoptosis of epithelial basal cells [16]. Patients with lichen planus experience a stressful event before the onset of the disease and major stressful events can exacerbate lichen planus. Psychological stress can trigger autoimmune disorders or skin disorders through neuroendocrine and neuroimmune dysregulation [17]. Immunological abnormalities lead to delayed growth of mucosa epithelial, which is responsible for hyperkeratosis [18].

From the higher psychological test scores in patients with OLP, it is not possible to determine whether the observed psychological changes are a direct cause of OLP or a consequence of OLP and its lesions. OLP patients who have a susceptibility to stress are also often concerned about the possibility of malignancy, which may contribute to the further potentiation of anxiety. OLP patients also report a poorer sleep quality due to a painful condition in their mouth that increases depression [19]. Therefore, OLP patients with psychological stress require a combination of OLP therapy and psychotherapy. OLP can be improved by regulating mood through medication and psychological counseling [20].

All the cases were treated with corticosteroids. Corticosteroids can be administered systemically or topically. The dose of corticosteroids was gradually reduced based on the severity of the lesion. Corticosteroids are endogenous steroid hormones produced from the adrenal cortex. Corticosteroids are also synthetically produced hormones used for pharmacotherapy. Corticosteroids have been widely used as anti-inflammatory drugs to combat autoimmune disorders. Systemic corticosteroids work by affecting lipid, carbohydrate, protein, calcium, and electrolyte. Glucocorticoids suppress all types of inflammation and allergic reactions by inhibiting the activity of plasminogen and reducing the inflammatory mediators such as prostaglandins and leukotrienes [21, 22].

The release of glucocorticoid compounds would increase the levels of corticotrophin-releasing hormone (CRH) and arginine vasopressin (AVP). Both of these hormones will activate the sympathetic nervous system and the hypothalamic-pituitary-adrenal (HPA) axis. Moreover, the hippocampus and amygdala, which regulate specific stress responses, also have glucocorticoid receptors. The translocation of steroid receptor complexes to the nucleus and the presence of glucocorticoid receptors can alter gene transcription. These changes will produce neurotransmitters including dopamine, serotonin, and neuropeptides, namely somatostatin and beta-endorphins [22, 23]. Indirectly, the release of endogenous glucocorticoids can regulate psychological stress responses such as depression and anxiety.

The limitation of this study is that the number of patients is too small so that the results cannot be generalized. Further longitudinal studies using larger samples need to be carried out.

## 4. Conclusion

Psychological stressors may play an essential role as a risk factor in the development of OLP. Therefore, dentists should consider the mental problems of OLP patients. This report may psychological interventions are a vital component of the collaborative care approach in the treatment of OLP.

## Figures and Tables

**Table 1:** DASS test results.

Case number	Depression		Anxiety		Stress	
	Before treatment	After treatment	Before treatment	After treatment	Before treatment	After treatment
1	Moderate	Mild	Severe	Mild	Mild	Mild
2	Severe	Moderate	Severe	Mild	Mild	Mild
3	Moderate	Moderate	Severe	Mild	Mild	Normal
4	Severe	Moderate	Moderate	Mild	Moderate	Mild
5	Mild	Normal	Moderate	Mild	Mild	Normal
6	Mild	Mild	Moderate	Mild	Mild	Mild

**Table 2:** The characteristic of the patients, clinical profile of OLP, and the treatment.

Case number	Sex	Age (years old)	Location	Type of OLP	Treatment
1	Male	42	Lip, dorsum of the tongue, right and left buccal mucosa	Reticular, erosive, and ulcerative	Systemic corticosteroid
2	Male	31	Lower labial mucosa, right and left buccal mucosa	Reticular	Systemic corticosteroid
3	Female	23	Right and left buccal mucosa, right and left side of the tongue	Reticular and erosive	Systemic corticosteroid
4	Female	32	Dorsum of the tongue, right and left buccal mucosa	Reticular and plaque-like	Topical corticosteroid
5	Female	54	Upper and lower labial mucosa, right and left buccal mucosa	Reticular and erosive	Systemic corticosteroid
6	Female	57	Lip, gingiva and vestibule of mandibular, right and left buccal mucosa	Reticular and erosive	Systemic corticosteroid

## Data Availability

The data used to support the findings of this case series are included in the article.
